# A systematic review of measures of shoulder pain and functioning using the International classification of functioning, disability and health (ICF)

**DOI:** 10.1186/1471-2474-14-73

**Published:** 2013-02-28

**Authors:** Yngve Roe, Helene Lundegaard Soberg, Erik Bautz-Holter, Sigrid Ostensjo

**Affiliations:** 1 Faculty of Health Sciences, Oslo and Akershus University College of Applied Sciences, Postboks 4 Street Olavs plass, Oslo 0130, Norway; 2 Department of Physical Medicine and Rehabilitation, Oslo University Hospital Ulleval, Oslo, Norway; 3 Faculty of Medicine, University of Oslo, Oslo, Norway

**Keywords:** ICF, Outcome assessment (health care), Shoulder pain, Shoulder, Health, Cross-sectional studies, *Disability evaluation, World health organization, Recovery of function, *Rehabilitation

## Abstract

**Background:**

Shoulder pain is a common condition with prevalence estimates of 7–26% and the associated disability is multi-faceted. For functional assessments in clinic and research, a number of condition-specific and generic measures are available. With the approval of the ICF, a system is now available for the analysis of health status measures. The aims of this systematic literature review were to identify the most frequently addressed aspects of functioning in assessments of shoulder pain and provide an overview of the content of frequently used measures.

**Methods:**

Meaningful concepts of the identified measures were extracted and linked to the most precise ICF categories. Second-level categories with a relative frequency above 1% and the content of measures with at least 5 citations were reported.

**Results:**

A set of 40 second-level ICF categories were identified in 370 single-item measures and 105 multi-item measures, of these, 28 belonged to activities and participation, 11 to body functions and structures and 1 to environmental factors. The most frequently addressed concepts were: pain; movement-related body functions and structures; sleep, hand and arm use, self-care, household tasks, work and employment, and leisure. Concepts of psycho-social functions and environmental factors were less frequently included. The content overview of commonly used condition-specific and generic measures displayed large variations in the number of included concepts. The most wide-ranging measures, the DASH and ASES were linked to 23 and 16 second-level ICF categories, respectively, whereas the Constant were linked to 7 categories and the SST and the SPADI to 6 categories each.

**Conclusions:**

This systematic review displayed that measures used for shoulder pain included more than twice as many concepts of activities and participation than concepts of body functions and structures. Environmental factors were scarcely addressed. The huge differences in the content of the condition-specific multi-item measures demonstrates the importance of clarifying the content to select the most appropriate measure both in research and in clinical work. For clinical situations, we propose use of a wide-ranging condition-specific measure that conceptualizes assessments of shoulder pain from a bio-psycho-social perspective. Further research is needed to assess how patient-reported problems in functioning are captured in the commonly used measures.

## Background

Shoulder pain is common in the general population; prevalence estimates range from 7 to 26 per cent [1]. The large range in the prevalence rates has been explained by the use of different definitions of the condition in the literature [1]. Pain in the neck or shoulder emerged as the most frequent work-related health complaint in a Norwegian cohort study, and diagnosed shoulder pain accounted for almost 18 per cent of all sick leave benefit claims in a Swedish survey [2,3]. Shoulder pain is characterised by restricted and painful movement of the arm, which results in difficulties in performing movement-related activities [4-6]. In recent decades, research has shown that psychological and social functioning may also be affected by shoulder pain; additionally, environmental factors may contribute to the development or persistence of the condition [7-10].

Functional assessments are an important aspect of clinical decision making and research pertaining to patients with shoulder pain. A number of condition-specific measures are available for making these assessments, including standardised clinical examination methods, patient-reported questionnaires and composite scores [5,6,11-14]. Whether the condition-specific symptoms should be limited to movement-related functions of the shoulder region or be expanded to include additional aspects of functioning, such as work, leisure activities and sleep quality has been debated [12,15]. To make the assessments more comprehensive and to facilitate comparisons with other health conditions, some have advocated the inclusion of generic measures in the assessments [7,13,16]. Generic measures may focus on a specific function or broadly include the concept of general health [12]. So far, there are no commonly accepted guidelines for functional assessment in the area of shoulder pain. Given the increasing standards of health measurements, considerable research effort has been devoted to investigating the psychometric properties of the condition-specific measures [17-24]. Although the content of such measures also needs to be considered, it often receives less attention [25].

With the approval of the International Classification of Functioning, Disability and Health (ICF) in 2001, a conceptual framework and classification is now available for content analysis of functional measures from a bio-psycho-social perspective [26]. The ICF is based on an integrative model that classifies functioning within the components of *body functions* (b), *body structures* (s), *activities & participation* (d) and *environmental* (e) and *personal factors* (not classified). The ICF classification provides categories of functioning and environmental factors that are arranged in a hierarchical fashion using an alphanumeric coding system. The initial letter refers to the component. This letter is followed by a numeric code that starts with the chapter number (e.g., Mobility, d4), which is followed by the second level (e.g., d445 Hand and arm use) and then the third level (e.g., d4452 Reaching). A fourth level of classification is also available when appropriate. The categories at a lower level are included in the higher level categories and chapters. Procedures have been established to classify the content of functional measures using ICF categories, regardless of their purpose, their extent and administration method [27,28].

The ICF classification is comprehensive. Shorter lists of categories, known as ICF core sets, have been developed to describe the typical spectrum of problems in the functioning of patients with a specific health condition [29]. The core set development process was based on literature reviews, expert surveys and single quantitative and qualitative clinical studies. A review investigating commonalities across ICF core sets for musculoskeletal conditions found a large number of common categories for the conditions low back pain, osteoarthritis, osteoporosis, and rheumatoid arthritis; however, there were also unique categories associated with each particular condition [30]. As part of this core set development process, a literature review was conducted to analyse the content of measures for each of the musculoskeletal disorders [31]. Such a review based on a bio-psycho-social perspective on functioning has not been conducted for shoulder pain. The aims of this systematic literature review were to identify the most frequently addressed aspects of functioning in assessments of shoulder pain and provide an overview of the content of frequently used measures.

## Methods

### Design

**Figure 1:**
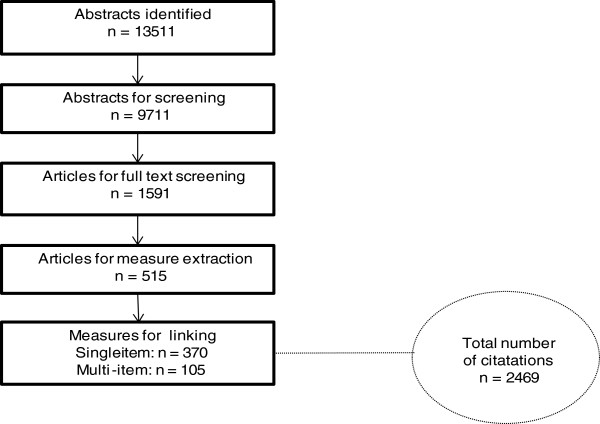
Flow chart of the literature search with the total number of identified measures and their number of citations.

A systematic literature review and content analysis of measures used in shoulder pain. The steps of the screening and extraction of measures are displayed in Figure 1.

### Literature search

The inclusion criteria were articles written in English, published in peer-reviewed journals and based on clinical studies on patients having shoulder pain. A highly sensitive 15-step search strategy for Medline was developed (Additional file 1) [32]. The Medline strategy was also adapted to Embase, PeDro, Cinahl and Central. The search was limited to studies published between January 2005 and May 2010. In a first step MeSH-terms related to shoulder pain were exploded and combined using the Boolean operator “OR”. Terms used for functional assessments were also combined with the Boolean operator “OR”. In the next step the MeSH-terms and the functional assessment terms were combined using the Boolean operator “AND”.

Articles based on studies of fractures, joint replacement, complete dislocation, malignant condition, rheumatic diagnosis and stroke were excluded, as were studies based exclusively on laboratory parameters or on a non-human population. The following designs or types of studies were also excluded: comments, letters, editorials, guidelines, conference reports, literature reviews, primary prevention studies, phase I or II studies, ecologic and economic evaluations, quantitative studies with less than 31 participants and studies on children.

### Screening and extraction of measures

All retrieved articles from the databases were imported to the same Endnote library (version X3, Thomson Reuters 1500 Spring Garden Street, Philadelphia) and screened for duplicates. In cases of multiple publications, the journal with the highest impact factor was selected. All remaining articles were imported into a Microsoft Access database (Microsoft Office 2003) for the abstract screening. Articles meeting any exclusion criteria were excluded. In cases where the decision was to include the article or the exclusion decision was ambiguous, full versions of the articles were retrieved. All abstracts were screened by one reviewer (YR); a random selection of 20% was also screened by a second reviewer (SO) before a final decision was made. Another predesigned Access database was used for the full version screening and extraction of measures. Where there was doubt as to which version of a measure had been used, a decision was made using the references given in the methods section of an article.

Information on nationality using the address of the first author, study design and types of interventions was recorded. The extracted measures were categorised as either single-item or multi-item measures. Single-item measures contained only one item, such as imaging and clinical tests and single questions on different domains; in contrast, multi-item measures included more than one test and question, such as different questionnaires and scales.

### Analyses

The content of the measures was linked to the ICF according to established rules [27,28]. Meaningful concepts were extracted and linked to the most specific ICF category possible. Items could contain more than one concept; for example, *I cannot lie on my right side at night because of my shoulder* contains the meaningful concepts *lie on my side* and *because of my shoulder*. The former was linked to the *maintaining a lying position* (d4150) and the latter to the *pain in upper limb* (b28014). For concepts not sufficiently specified to be linked, the *non-definable* option was chosen. If a concept was not covered by the ICF classification, the option *not covered* was chosen [27,28]. All measures were linked by one reviewer (YR) and a random selection of twenty-five per cent of the multi-item measures were also linked by a second reviewer (SO). The single-item measures were discussed with a clinician and researcher experienced in rehabilitation of shoulder pain (KE). The ICF links of ten measures that had already been published in scientific journals or were available from previous reviews performed by the ICF Research Branch were accepted for use in the current study [33,34].

Relative frequencies of the linked second-level ICF categories for each component were estimated from the total number of citations. Only ICF categories that arose with a frequency of at least 1% are presented. A frequency of 10% was chosen as the arbitrary cut off to classify a category as high frequent. In cases where concepts were linked to a third- or fourth-level category, they were aggregated to the second level. For example, a concept linked to the third-level category *turning or twisting the hands or arms* (d4453) was reported under the second-level *hand and arm use* (d445) category. When an ICF category was assigned repeatedly in the same measure, it was only counted once. Moreover, the content of measures cited in at least 5 different articles were presented at the ICF chapter level and more detailed in the Additional file 2.

Reliability of the abstract screening and linking procedures were measured with percentage agreement and estimation of Cohen’s Kappa coefficient. The 95% confidence intervals for the Kappa coefficient were constructed using the bias-corrected percentile method [35,36]. A Kappa coefficient of 0–0.4 was considered poor, 0.41 – 0.60 fair to good and 0.61 – 1.00 excellent [37]. The agreement in the counter-screening of abstracts between reviewers was 87.3%. The estimated Kappa coefficient was 0.62 (95% CI, 0.59 - 0.66), which is considered good or excellent. The agreement in the linking procedure between reviewers was 80.8%. The estimated Kappa coefficient was 0.81 (95% CI, 0.77 - 0.85), which was classified as excellent.

## Results

### Literature search

A total of 13,511 articles were identified through the literature search; of these articles, 1591 full versions were screened, and 515 were included. Altogether 475 different measures were extracted with a total of 2469 citations. Among them, 370 were single-item measures and 105 were multi-item measures. A total of 20,517 meaningful concepts were extracted from the measures, of which 86.3% were linked to the ICF. The share of concepts that were not covered or not definable was 13.7%. The procedure is displayed in Figure 1.

### Study characteristics

According to nationality, Europe accounted for 44% of the articles, Canada and USA for 32% and Asia for 15%. Approximately 9% of the articles were from other continents. Sixty per cent of the articles contained studies with an interventional design (e.g., randomised controlled trial or case control trial), while thirty-nine per cent of articles were based on an observational study (longitudinal or cross-sectional). Only a single article based on a qualitative study was present in the sample. Ninety-one per cent of the articles included participants with a diagnosed shoulder condition, of whom 52% were diagnosed with subacromial pain conditions, 17% with instability or SLAP-lesions, 9% with adhesive capsulitis, 18% with mixed diagnoses and 4% with other diagnoses. Nine per cent of the articles included individuals with self-reported shoulder conditions only.

### Second-level ICF categories linked to concepts contained in the measures

A total of 40 second-level ICF categories with a frequency above 1% were identified in the components of *body functions and structures*, *activities and participation* and *environmental factors*.

Eleven second-level ICF categories were identified within the *body functions and structures* component, as shown in Table 1. Of these, five categories were located in the *neuromusculoskeletal or movement related functions* (b7) chapter, three in *mental functions* (b1), two in *sensory functions and pain* (b2) and one in *structures related to movements* (s7). The five second-level categories with a relative frequency above 10% were *sensation of pain* (b280), *mobility of joint functions* (b710), *structure of shoulder region* (s720), *muscle power functions* (b730) and *sleep functions* (b134).

**Table 1:** Relative frequency (%) of second level ICF categories linked to the concepts contained in the measures for the ICF component body functions and structures (n= 2469) in ranked order

**ICF second level categories (n=11)**		**(%)**
b280	Sensation of pain	47,3
b710	Mobility of joint functions	34,7
s720	structure of shoulder region	24.9
b730	Muscle power functions	24,2
b134	Sleep functions	17,5
b715	Stability of joint functions	7,1
b152	Emotional functions	6,3
b780	Sensations related to muscles and movement functions	3,3
b130	Energy and drive functions	3,1
b265	Touch function	2,3
b720	Mobility of bone functions	2,1

As displayed in Table 2, 28 second-level ICF categories were identified within the *activities and participation* component. Of these, eight categories had a relative frequency above 10%. Nine categories belonged to the *mobility* chapter (d4), six to *self-care* (d5*)*, four to *domestic life* (d6), three to *interpersonal interactions and relationships* (d7) and *major life areas* (d8), and one category each to the chapters of *community, social and civic life* (d9)*, learning and applying knowledge* (d1) *and general tasks and demands* (d2). The eight categories with a frequency above 10% were, in ranked order: *hand and arm use* (d445), *remunerative employment* (d850), *recreation and leisure* (d920), *lifting and carrying objects* (d430), *washing oneself* (d510), *dressing* (d540), *caring for body parts* (d520) and *doing housework* (d640).

**Table 2:** Relative frequency (%) of second level ICF categories linked to the concepts contained in the measures for the ICF component activities and participation (n= 2469) in ranked order

**ICF second level categories (n=28)**		**(%)**
d445	Hand and arm use	24,5
d850	Remunerative employment	23,2
d920	Recreation and leisure	18,3
d430	Lifting and carrying objects	17,1
d510	Washing oneself	17
d540	Dressing	15,8
d520	Caring for body parts	12,7
d640	Doing housework	10,4
d415	Maintaining a body position	6
d230	Carrying out daily routine	4,5
d475	Driving	4,7
d530	Toileting	3,6
d650	Caring for household objects	3,6
d620	Acquisition of goods and services	3,4
d470	Using transportation	3,6
d760	Family relationships	3
d550	Eating	2,9
d450	Walking	2,8
d410	Changing basic body position	2,6
d630	Preparing meals	2,6
d750	Informal social relationships	2,6
d455	Moving around	2,5
d770	Intimate relationships	2,3
d859	Work and employment, other specified and unspecified	2,2
d170	Writing	2,1
d440	Fine hand use	2,1
d570	Looking after one’s health	1,1
d820	School education	1

In the ICF component of *environmental factors*, the only identified second-level category was *products or substances for personal consumption* (e110). This category which was located in the *products and technology* (e1) chapter had a relative frequency of 8.8%.

### Distribution of ICF codes within the measures

The 16 condition-specific and 7 generic multi-item measures with five or more citations are displayed in Table 3. By far the most cited were Constant-Murley Shoulder Score (Constant) (124 citations), followed by the American Shoulder and Elbow Surgeons standardized form for assessment of the shoulder (ASES) (77 citations), the University of California at Los Angeles shoulder rating scale (UCLA) (64 citations) and the Disability of the Arm, Shoulder and Hand scale (DASH) (51 citations). All of the condition-specific measures included categories from both the *body functions and structures* and *activities and participation* components of the ICF. Of these, the DASH and ASES were the most wide-ranging, containing meaningful concepts linked to categories in 11 and 9 chapters, respectively. By contrast, the Shoulder Pain and Disability Index (SPADI) and the Walch-Duplay Score only contained categories belonging to three ICF chapters. The most-frequently cited generic measure, the MOS 36-item short-form health survey (SF-36) (46 citations), was linked to seven chapters: two of which were in the *body functions and structures* component, and five of which were in the a*ctivities and participation* component.

**Table 3:** Number of citations and content overview at ICF chapter-level of the most frequently identified multi-item measures

**Cond-spec. measures (n=16)**	**Number of citations**	**Mental functions (b1)**	**Sensory functions and pain (b2)**	**Neuromuscular and movement (b7)**	**Structures related to movement (s7)**	**Learning and applying knowledge (d11)**	**General task and demands (d2)**	**Mobility (d4)**	**Self-care (d5)**	**Domestic life (d6)**	**interpersonal interactions and rel. (d7)**	**Major life areas (d8)**	**Community, social and civic life (d9)**	**Products and technology(e1)**	**Natural environment and hum. ch. (e2)**	**Support and relationships (e3)**
Constant	124	√	√	√				√				√	√			
ASES	77	√	√	√	√			√	√			√	√	√		
UCLA	64		√	√				√	√	√				√		
DASH	51	√	√	√		√	√	√	√	√	√	√	√			
SST	46	√	√					√	√			√				
Rowe	31		√	√				√	√			√	√	√		
SPADI	31		√					√	√							
WORC	21	√	√	√				√	√	√		√				
SRQ	15	√	√					√	√	√		√	√			
SDQ	14	√	√					√	√							
OSS	11	√	√					√	√	√		√				
WOSI	8	√	√	√			√	√	√	√	√	√				
QuickDASH	7	√	√	√			√	√	√	√	√	√				
FLEX-SF	6			√				√	√	√		√	√			
Penn	5		√	√									√			
Walch-Duply	5		√	√									√			
**Generic measures (n=7)**																
SF-36	46	√	√				√	√		√		√	√			
SF-12	9	√						√				√				
JCQ	8							√			√	√			√	√
Nordic	7		√									√				
EQ-5D	6	√	√													
FABQ	5		√									√				
4DSQ	5	√	√					√								

Constant = the Constant Murley shoulder score [5], ASES = the American Shoulder and Elbow Surgeons standardized form for assessment of the shoulder [6], UCLA = the University of California at Los Angeles shoulder rating scale [38], DASH = the Disability of the Arm, Shoulder and Hand scale [39], SST = the Simple Shoulder Test [40], SPADI = the Shoulder Pain and Disability Index [41], Rowe = a Rating sheet for Bankard repair [42], WORC = the Western Ontario Rotator Cuff Index [43], SRQ = the Shoulder Rating Questionnaire [44], SDQ = the Shoulder Disability Questionnaire [45], OSS = the Oxford Shoulder Score [46], WOSI = the Western Ontario Shoulder Instability Index [47] , QuickDASH = the shortened disabilities of the arm, shoulder and hand questionnaire [48], FLEX-SF = the Flexilevel Scale of Shoulder Function [49], Penn = the Penn shoulder score [50] , the Walch-Duplay shoulder score [51] , SF-36 = the MOS 36-item short-form health survey [52] , SF-12 = a 12-Item Short-Form Health Survey [53], JCQ = the Job Content Questionnaire [54], Nordic = the standardized Nordic questionnaires for the analysis of musculoskeletal symptoms [55], EQ-5D = a measure of health status from the EuroQol Group [56], FABQ = a Fear-Avoidance Beliefs Questionnaire [57], 4DSQ = the Four-Dimensional Symptom Questionnaire [58].

Of the condition-specific measures, the ASES, UCLA and the Rating Sheet of Bankard repair (Rowe) also included concepts that were linked to an environmental factor, all of which belonged to the *products and technology* (e1) chapter. Only one of the generic measures, the Job Content Questionnaire (JCQ), included environmental factors. Its content was linked to two chapters other than *products and technology (*e1); specifically, it was also linked to the *natural environment and human-made changes to environment* (e2) and s*upport and relationships* (e3) chapters.

The most comprehensive measure of *mental functions* (b1) was the generic Four-Dimensional Symptom Questionnaire (4DSQ). It includes concepts linked to five second-level categories: *consciousness functions* (b110), *energy and drive functions* (b130), *sleep functions* (b134), *emotional functions* (b152) and *higher-level cognitive functions* (b164). The SF-36 had concepts linked to two mental function categories: the *energy and drive functions* (b130) and *emotional functions* (b152). Of the condition-specific measures, none of the most cited contained other mental functions than *sleep functions* (b134). The UCLA (the third most cited) did not address any *mental functions* (b1) concepts. Looking at employment and leisure activities, the content of 11 of the 16 condition-specific measures was linked to *remunerative employment* (d850), eight to *recreation and leisure* (d920) and seven of the measures to both ICF categories. The UCLA, SPADI, the Shoulder Disability Questionnaire (SDQ) and the Flexilevel Scale of Shoulder Function (FLEX-SF) contained no concepts related to work and leisure. Of the seven generic measures, five included work functions; only one, the SF-36, asked for information about leisure activities.

The 28 condition-specific and 7 generic single-item measures with five or more citations are displayed in Table 4. Patient-reported shoulder pain intensity was the most frequently cited (200 citations) followed by active range of motion (170 citations), Magnetic Resonance Imaging (MRI/MRA) (125 citations), muscle strength (98 citations), X-ray (81 citations), passive range of motion (61 citations) and ultrasonography (57 citations). The measures contained concepts that were linked to categories in three ICF chapters of the *body functions and structures* component: *sensory functions and pain* (b2), *neuromusculoskeletal or movement related functions* (b7) and *structures related to movements* (s7). By contrast, the generic single-item measures were (with one exception) linked to categories of *activities and participation* or *environmental factors*. These categories belonged to the *self-care* (d5*)*, *major life areas* (d8), *community, social and civic life* (d9) and *products and technology* (e1) chapters. Two measures that requested the use of medication or smoking habits were the only concepts of environmental factors among the single-item measures.

**Table 4:** Number of citations and content overview at ICF chapter-level of the most frequently identified single-item measures

**Cond-spec. measures (n=28)**	**Number of citations**	**Mental functions (b1)**	**Sensory functions and pain (b2)**	**Neuromuscular and movement (b7)**	**Structures related to movement (s7)**	**Self-care (d5)**	**Major life areas (d8)**	**Community, social and civic life (d9)**	**Products and technology (e1)**
Patient-report pain intensity	200		√						
Active range of motion	170			√					
Magnetic Resonance Imaging (MRI/MRA)	125				√				
Muscle strength	98			√					
X-ray	81				√				
Passive range of motion	61			√					
Ultrasonography	57				√				
Hawkins-Kennedy test	47		√	√					
Neer test	41		√	√					
Painful arc	27		√	√					
Apprehension test	25		√	√					
Resisted isometric abduction	22		√	√	√				
Arthroscopic examination of the shoulder	18				√				
Active compression test (O’Brian)	17		√	√	√				
Lift-off test	16		√	√	√				
Speed test	15				√				
Impingement signs	13		√						
Electromyelography (EMG)	12			√					
Relocation test (Jobe relocation)	10		√	√					
Yergason test	10			√	√				
Palpation sensitivity rotator cuff/biceps	9		√						
Empty can test	9			√	√				
Sulcus sign	8			√	√				
Jobe test for supraspinatus (Fulcrum’s test)	8			√	√				
Belly press test	6		√	√	√				
Compression-rotation test	5			√	√				
Instability testing shoulder	5			√					
Drop arm test	5		√	√	√				
**Generic measures (n=7)**									
Work absenteism	31						√		
Medication	15								√
Smoking habits	14								√
Sport activity	17							√	
Comb hair	7					√			
Physical activity	7							√	
Sleep quality	5	√							

## Discussion

Using the ICF as a reference, we first identified and quantified the concepts included in frequently used measures of shoulder pain and functioning. The content of the measures was linked to 11 different ICF categories within 3 of 8 domains of *body functions and structures*, and 28 ICF categories within 8 of 9 domains of *activitie*s *and participation*. *Environmental factors* were scarcely addressed, accounting for only one category. The finding displays that the measures of shoulder pain cover a large number of concepts of daily activities and also some particular concepts of body functions.

As expected, the ICF category *sensation of pain* was highest ranked. Different concepts of pain were requested in both condition-specific single and multi-item measures and also in generic measures. This is consistent with previous recommendations to regard pain as a global construct measured by pain intensity and by interference with activities [59]. In a systematic literature review on prognostic factors in primary care populations of shoulder disorders, strong evidence was found that high pain intensity at baseline predicts a poor outcome [60]. The ICF categories *mobility of joint*, *structures of the shoulder region* and *muscle power functions* were ranked second, third and fourth, and in most cases linked from concepts in condition-specific measures. However, not all such concepts were common in the measures; the ICF category *muscle endurance* was not frequent above the 1% limit, although isometric muscle endurance has been proposed as a psycho-physiological measure for shoulder pain [61].

*Sleep functions*, classified in the ICF as a mental function, was the fifth most frequent ICF category. Concepts of sleep were included in many condition-specific and generic measures, whereas concepts linked to the less frequent ICF categories *emotional functions* and *energy and drive* were extracted from only a few measures. A study that included a community based population of subjects with chronic shoulder pain, found that the relation between pain and psychological health was dependent of level of disability [9]. Moreover, a previous review points to the influence of psychosocial and behavioural factors in chronic neck-and-shoulder pain [62]. According to the current finding, concepts of psychological health may be underestimated in commonly used measures of shoulder pain. However, one comprehensive measure on psychological functioning was found, the generic 4DSQ, which captured five different *mental functions* according to the ICF.

Several of the predominant concepts in measures of shoulder pain and functioning, were in the *activities and participation* component. Ten ICF categories belonged to *mobility functions* and five each to *self-care* and *domestic life*. *Hand and arm use* and *lifting and carrying* were both among the five highest ranked activities and participation categories. Concepts linked to these two ICF categories were extracted from almost all the condition-specific multi-item measures (see Additional file 2). This demonstrates that task orientated movements of the upper-extremity is in the core of the assessment of shoulder pain. The high ranking of the ICF category *remunerative employment*, was consistent with the high numbers reporting work-relatedness of their shoulder disorder in a previous epidemiological study [2]. Work-related concepts were addressed in a majority of the multi-item condition-specific measures, although the UCLA, SPADI and SDQ did not address any concepts of work. In a recent review of concepts in vocational rehabilitation measures, a number of work-related concepts were extracted [63]. One of the commonly used vocational measures, the JCQ was also identified in the current review [54]. Its comprehensiveness indicates that assessments of work need to capture several different functional domains.

Previous research shows that also social functioning may be affected by shoulder pain [7-10]. *Family-*, *informal social-* and *intimate relationship*, all appeared among the lower ranked ICF categories and these concepts were included in only one condition-specific measure, the DASH. Although the SF-36 contains a social subscale, none of its concepts were linked to the ICF category *interpersonal interactions and relationships*[33]. This indicates that the SF-36 requests social relationships in a more general way and not as specific interpersonal interactions.

*Products or substances for personal consumption* that appeared with a relative frequency of 8.8%, was the only environmental factor above the 1% criteria. This finding reflects that the impact of the environment on functioning is not sufficiently taken into consideration in the assessments of shoulder pain. According to the ICF, the environment contains a large number of physical, social and attitudinal factors which may limit or facilitate functioning. Although some previous research has been devoted to identify risk factors in the workplace environment, the significance of external factors has scarcely been addressed within the shoulder pain research [64].

Concepts measured in different musculoskeletal disorders were identified in a previous review, and of particular interest for the current study was low back pain [31]. Although there were large similarities between the content of the shoulder pain and low back pain measures, some differences emerged. The comparisons showed that the measures of shoulder pain contained a higher number of concepts within self-care and domestic life, whereas the low back pain measures contained a higher number of environmental factor concepts, concerning support and relationships to persons and the attitudes of health professionals.

This review identified 44 condition-specific and 15 generic measures in use to assess functioning in patients with shoulder pain. When comparing the content of the single- and multi-item measures we found that the former requested only pain and movement related functions, whereas the latter included a wide range of body functions and structures, and activities. The wide-ranging DASH and the ASES were linked to 23 and 16 ICF categories respectively, whereas the Constant was linked to 7 categories and the Simple Shoulder Test (SST) and SPADI to 6 categories each (see Additional file 2). These comparisons, using the ICF as a framework, disclose both the similarities and differences in content of measures that all aim to assess aspects of functioning in patient with shoulder pain.

The variation in the type and number of concepts in the condition-specific measures might reflect disparate views on disability among developers of measures. Some of the measures, such as the SPADI and the Oxford Shoulder Score (OSS) were developed to capture joint-specific concepts and to avoid the influence of co-morbidity [41,65]. On the contrary, the DASH aims at capture disability, defined as difficulty in doing activities in any domain of life [39]. Due to the complexity of the disability of shoulder pain, and the narrow content of many condition-specific measures, it has been recommended to supplement the condition-specific measures with the generic SF-36 [7,13,16]. However, as demonstrated in the current study, the SF-36 includes few additional concepts to those requested in the most wide-ranging condition-specific measures. Clarifying the content is of great importance for selecting the most appropriate measures in clinical work and in research, although the choice of a measure is also dependent on the purpose, patient population and the psychometric properties. In our opinion, use of a wide-ranging condition-specific measure may enhance the quality of assessments in many clinical situations. The wide-ranging (Quick-) DASH and the ASES were found to be among the most extensively investigated measures according to measurement properties in a recent review [24].

The current review had some limitations that should be noted. Meaningful concepts in the measures referring to *personal factors* in the ICF, such as fear avoidance and coping strategies were not reported. The updated linking rules enable the identification of *personal factors*, but they are still not classified in the ICF [28]. For 10 measures identified in the study, the content was linked in previous studies (32, 32). The commonly used SF-36 was analysed using the first version of the ICF linking rules [27]. Use of the updated linking rules may have given a somewhat different result [28]. For interpretation of the results, it is of importance that a particular ICF category was reported only once for each measure. As such, the content overview of the measures provides information on the breadth of each measure rather than their depth.

## Conclusions

Using the ICF as a reference, a total of 40 second-level categories was used to classify the content of condition-specific and generic measures of shoulder pain. The most frequently addressed concepts were pain, movement-related body functions and structures, sleep, hand and arm use; self-care, household tasks, work and employment, and leisure activities. Concepts of psycho-social functioning and environmental factors were less frequently addressed. Commonly used condition-specific measures showed a large variation in content; the DASH and the ASES were linked to more than twice as many ICF categories as the Constant, SST and SPADI. These large differences demonstrate the importance of clarifying the content to select the most appropriate measure both in research and in clinical work. For clinical situations, we propose use of a wide-ranging condition specific measure that conceptualizes assessments of shoulder pain from a bio-psycho-social perspective. Further research is needed to investigate whether patient-reported problems in functioning are captured in the commonly used condition-specific and generic measures.

## Competing interests

The authors declare that they have no competing interests.

## Authors’ contributions

YR, HLS, EB-H and SO participated in the planning and design of the study. YR developed a search strategy and collected the data. YR and SO participated in the screening and linking. YR, SO, HLS and EB-H drafted the manuscript. All authors read and approved the manuscript.

## Pre-publication history

The pre-publication history for this paper can be accessed here:

http://www.biomedcentral.com/1471-2474/14/73/prepub

## Supplementary Material

Additional file 1 Final search strategy for Medline. Click here for file

Additional file 2 Overview of second-level ICF categories in the most common multi-item measures. Click here for file
