# Supplementary material for: A systematic review of measures of shoulder pain and functioning using the International classification of functioning, disability and health (ICF)
Source: BMC Musculoskelet Disord. 2013 Feb 28;14:73. doi: 10.1186/1471-2474-14-73 (PMC3668165; doi:10.1186/1471-2474-14-73)
Supplement: Additional file 1 — Final search strategy for Medline. [file 1471-2474-14-73-S1.pdf]

Take a look at the new OvidSP Try it now!

Close X

Wolters Kluwer Health | OvidSP

Current Search Results | Main Search Page | Support & Training | Help | Logoff

Current Search History

Ovid MEDLINE(R) In-Process & Other Non-Indexed Citations and Ovid MEDLINE(R) 1950 to Present

#	Searches	Results	Search Type
1	shoulder dislocation/ or shoulder/ or shoulder impingement syndrome/ or shoulder fractures/ or shoulder pain/ or shoulder joint/ or acromioclavicular joint/ or thoracic outlet syndrome/ or scapula/ or acromion/ or rotator cuff/ or Upper Extremity/ or bursitis/ [mp=title, original title, abstract, name of substance word, subject heading word, unique identifier]	33695	Advanced
2	(shoulder$ or acromio$ or acromia$ or humeroscapular or rotator cuff$).hw.	25883	Advanced
3	(shoulder$ or glenohumeral$ or adhesive capsulit$ or bursitis or rotator cuff$ or acromio$ or acromia$ or scapula$ or humeroscapular$ or humero scapular$ or glenohumeral$).ti.	19544	Advanced
4	(test$ or index$ or score$ or scale$ or survey$ or indicator$ or questionnaire$ or measure$ or examination$ or intervention$ or function$ or performance$ or rate or rating or disability or outcome).hw.	2425807	Advanced
5	(outcome or outcomes or test or tests or index$ or score$ or scale$ or survey$ or questionnaire$ or rate or rating).tw.	3174916	Advanced
6	"Diagnostic Techniques and Procedures"/	1526	Advanced
7	1 or 2 or 3	36024	Advanced
8	4 or 5 or 6	4552915	Advanced
9	7 and 8	11896	Advanced
10	(shoulder$ or glenohumeral$ or adhesive capsulit$ or bursitis or rotator cuff$ or acromio$ or acromia$ or scapula$ or humeroscapular$ or humero scapular$ or troracic outlet syndrome).tw.	41919	Advanced
11	in-data-review.st.	144337	Advanced
12	in-process.st.	200745	Advanced
13	pubmed-not-medline.st.	388762	Advanced
14	5 and 10 and (11 or 12 or 13)	678	Advanced
15	9 or 14	12316	Advanced

16	limit 15 to yr="2005 -Current"	6006	Advanced
17	limit 16 to (danish or english or norwegian or swedish)	5485	Advanced
18	limit 17 to animals	175	Advanced
19	limit 18 to humans	45	Advanced
20	18 not 19	130	Advanced
21	17 not 20	5355	Advanced
22	limit 21 to ("all child (0 to 18 years)" or "all aged (65 and over)") [Limit not valid in EMBASE; records were retained]	2464	Advanced
23	limit 22 to "all adult (19 plus years)" [Limit not valid in EMBASE; records were retained]	2139	Advanced
24	22 not 23	325	Advanced
25	21 not 24	5030	Advanced
26	limit 25 to (embryo <first trimester> or infant <to one year> or child <unspecified age> or preschool child <1 to 6 years> or school child <7 to 12 years> or aged <65+ years>) [Limit not valid in Ovid MEDLINE(R),Ovid MEDLINE (R) In-Process; records were retained]	5030	Advanced
27	limit 26 to adult <18 to 64 years> [Limit not valid in Ovid MEDLINE(R),Ovid MEDLINE(R) In-Process; records were retained]	5030	Advanced
28	26 not 27	0	Advanced
29	25 not 28	5030	Advanced
30	limit 29 to yr="2005"	778	Advanced
31	limit 29 to yr="2006"	713	Advanced
32	limit 29 to yr="2007"	925	Advanced
33	limit 29 to yr="2008"	1023	Advanced
34	limit 29 to yr="2009"	1175	Advanced
35	limit 29 to yr="2010"	416	Advanced
36	remove duplicates from 30	745	Advanced
37	remove duplicates from 31	703	Advanced
38	remove duplicates from 32	898	Advanced
39	remove duplicates from 33	995	Advanced
40	remove duplicates from 34	1109	Advanced
41	remove duplicates from 35	412	Advanced
42	36 or 37 or 38 or 39 or 40 or 41	4862	Advanced
43	(2005 jan or 2005 feb or 2005 mar or 2005 apr or jan 2005 or feb 2005 or mar 2005 or apr 2005).dp.	126971	Advanced
44	42 not 43	4684	Advanced
45	limit 44 to yr="2010"	412	Advanced
46	limit 44 to yr="2009"	1109	Advanced

47	limit 44 to yr="2008"	995	Advanced
48	limit 44 to yr="2007"	898	Advanced
49	limit 44 to yr="2006"	703	Advanced
50	limit 44 to yr="2005"	567	Advanced

Copyright (c) 2000-2010 Ovid Technologies, Inc.

By accessing or using OvidSP, you agree to Ovid's terms of use, conditions and all applicable laws. If you do not agree to these terms you may not use this Site.

Version: OvidSP_UI02.03.01_H11_2.101, SourceID 49793
