# Supplementary material for: A systematic review of measures of shoulder pain and functioning using the International classification of functioning, disability and health (ICF)
Source: BMC Musculoskelet Disord. 2013 Feb 28;14:73. doi: 10.1186/1471-2474-14-73 (PMC3668165; doi:10.1186/1471-2474-14-73)
Supplement: Supplementary file 2 [file 1471-2474-14-73-S2.docx]

**Cond-spec. measures**	Number of citations	Consciousness functions (b110)	Energy and drive functions (b130)	Sleep functions (b134)	Emotional functions (b152)	Higher-level cognitive functions (b164)	Touch function (b265)	Sensation of pain (b280)	Mobility of joint functions (b710)	Stability of joint functions (b715)	Mobility of bone functions (b720)	Muscle power functions (b730)	Sensations rel. to muscles and mov. (b780)	Structure of shoulder region (s720)	Writing (d170)	Carrying out daily routine (d230)	Changing basic body position (d410)	Maintaining a body position (d415)	Lifting and carrying objects (d430)	Fine hand use (d440)	Hand and arm use (d445)	Walking (d450)	Moving around (d455)	Using transportation (d470)	Driving (d475)	Washing oneself (d510)	Caring for body parts (d520)	Toileting (d530)	Dressing (d540)	Eating (d550)	Looking after one's health (d570)	Acquisition of goods and services (d620)	Preparing meals (d630)	Doing housework (d640)	Caring for household objects (d650)	Formal relationships (d740)	Informal social relationships (d750)	Family relationships (d760)	Intimate relationships (d770)	School education (d820)	Acquering, keeping and term. a job (d845)	Remunerative employment (d850)	Non-remunerative employment (d855)	Work and employment, other spec. (d859)	Recreation and leisure (d920)	Products and subst. for pers. cons. (e110)	Acquaintances, peers coll, neighb. (e325)	People in position of authority (e330)
Constant	124			√				√	√			√									√																					√			√			
ASES	77			√				√	√	√		√		√				√	√		√					√	√	√	√													√			√	√		
UCLA	64							√	√			√									√				√	√	√		√			√		√												√		
DASH	51			√			√	√			√	√	√		√	√			√		√			√		√	√		√	√			√	√	√		√	√	√			√			√			
SST	46			√				√											√		√					√																√						
Rowe	31							√	√	√		√							√		√						√															√			√	√		
SPADI	31							√										√	√		√					√			√																			
WORC	21			√	√			√	√			√	√						√		√						√		√		√			√	√							√						
SRQ	15			√				√											√		√				√	√	√		√					√						√		√	√		√			
SDQ	14			√	√													√	√	√	√			√	√	√			√																			
OSS	11			√				√											√	√	√			√	√	√	√		√			√		√								√						
WOSI	8			√	√			√	√	√		√	√						√																							√			√			
QuickDASH	7			√			√	√				√				√					√					√				√				√			√	√				√						
FLEX-SF	6								√								√		√	√	√					√	√		√																			
Penn	5			√														√	√	√	√					√	√		√				√	√								√			√			
Walch-Duplay	5							√	√	√																																			√			
**Generic measures**																																																
SF-36	46		√		√			√								√	√		√		√	√	√			√			√					√								√		√	√			
SF-12	9		√		√																		√																					√				
JCQ	8																	√	√																	√					√	√					√	√
Nordic	7							√																																		√						
EQ-5D	6				√			√																																								
FABQ	5							√																																		√						
4DSQ	5	√	√	√	√	√		√																√																								
